# Rural Tanzanian women's awareness of danger signs of obstetric complications

**DOI:** 10.1186/1471-2393-9-12

**Published:** 2009-03-26

**Authors:** Andrea B Pembe, David P Urassa, Anders Carlstedt, Gunilla Lindmark, Lennarth Nyström, Elisabeth Darj

**Affiliations:** 1 Department of Obstetrics and Gynecology, School of Medicine, Muhimbili University of Health and Allied Sciences, P. O. Box 65117, Dar es Salaam, Tanzania; 2 Department of Community Health, School of Public Health and Social Sciences, Muhimbili University of Health and Allied Sciences, P.O. Box 65015, Dar es Salaam, Tanzania; 3 International Maternal and Child Health, Department of Women's and Children's Health, Uppsala University, SE-751 85, Uppsala, Sweden; 4 Department of Surgery, Central Hospital, 65230, Karlstad, Sweden; 5 Department of Public Health and Clinical Medicine, Umeå University, SE-901 85, Umeå, Sweden

## Abstract

**Background:**

Awareness of the danger signs of obstetric complications is the essential first step in accepting appropriate and timely referral to obstetric and newborn care. The objectives of this study were to assess women's awareness of danger signs of obstetric complications and to identify associated factors in a rural district in Tanzania.

**Methods:**

A total of 1118 women who had been pregnant in the past two years were interviewed. A list of medically recognized potentially life threatening obstetric signs was obtained from the responses given. Chi- square test was used to determine associations between categorical variables and multivariate logistic regression analysis was used to identify factors associated with awareness of obstetric danger signs.

**Results:**

More than 98% of the women attended antenatal care at least once. Half of the women knew at least one obstetric danger sign. The percentage of women who knew at least one danger sign during pregnancy was 26%, during delivery 23% and after delivery 40%. Few women knew three or more danger signs. According to multivariate logistic regression analysis having secondary education or more increased the likelihood of awareness of obstetric danger signs six-fold (OR = 5.8; 95% CI: 1.8–19) in comparison with no education at all. The likelihood to have more awareness increased significantly by increasing age of the mother, number of deliveries, number of antenatal visits, whether the delivery took place at a health institution and whether the mother was informed of having a risks/complications during antenatal care.

**Conclusion:**

Women had low awareness of danger signs of obstetric complications. We recommend the following in order to increase awareness of danger signs of obstetrical complications: to improve quality of counseling and involving other family members in antenatal and postnatal care, to use radio messages and educational sessions targeting the whole community and to intensify provision of formal education as emphasized in the second millennium development goal.

## Background

Worldwide, in 2005, 535,900 women died from causes related to pregnancy and childbirth; half of these deaths occurred in sub-Saharan Africa [1]. Tanzania, located in sub-Saharan Africa has an estimated maternal mortality ratio ranging from 578 to 950 per 100,000 live-births [1,2]. The common causes of maternal deaths are hemorrhage, postpartum infection, hypertensive disorders, obstructed labor and abortion complications [3,4]. These life-threatening complications are treatable thus most of these deaths are avoidable if women with the complications have timely access to appropriate emergency obstetric care [5].

Three phases of delay to access care have been described [6]: delay in making the decision to seek care; delay in arrival at a health facility; and delay in receiving appropriate treatment after arriving at the health facility. Awareness of the danger signs of obstetric complications among pregnant women and in their communities is the first step to accepting appropriate and timely referral to essential obstetric and newborn care, thus, reducing the first and second phases of delay [5,7,8]. The danger signs occurring during pregnancy are predictive of poor outcome rather than historic risk factors [9].

In 2002, the Tanzanian government introduced focused antenatal care (FANC), a new model of antenatal care recommended by the World Health Organization [10]. Individual counseling on birth preparedness and complication readiness including danger signs of obstetric complications during antenatal and postnatal visits is emphasized. At present in the country, 94% of women attend antenatal care at least once, thus it is expected that majority of women would have received information on danger signs of obstetric complications [2]. Despite the high antenatal care attendance, a study in Rufiji showed that compliance with emergency referral advice is low. Half of the women with obstetric complications referred did not arrive at the referral hospitals [11]. Late or failure of women with obstetric complications to reach referral hospitals may be contributed by many reasons. One reason may be lack of awareness of significance of symptoms or obstetric complications.

The aim of this study was to assess women's awareness of danger signs of obstetric complications and to identify associated factors in a rural district in Tanzania. This information is necessary for service providers and district-health management teams for improving the quality of antenatal care services provided in both the first line and referral health institutions.

## Methods

### Study setting

This was a cross-sectional study undertaken in Rufiji district, between November and December 2006. Rufiji district is one of the six districts of Coastal Region in Tanzania. The district covers an area of about 14,500 km^2^. It has an estimated population of 203,000 with approximately 52% females, according to the 2002 Census [12]. The majority of the population are peasants, with 38% living below the national basic needs poverty line [13]. Geographically, the Rufiji district consists of flood plain, coastal-delta, and plateau zones. Most road networks in the district are difficult to pass especially during the rainy season. The district has five divisions divided into 19 wards: eight wards in the flood plain, four in the coastal-delta, and seven in the plateau zone. The total number of villages is 128, each with an average population of 1600.

The district has two hospitals, both providing comprehensive emergency obstetric care, four rural health centers and 48 dispensaries. Health workers provide maternal care in all health institutions.

### Sampling method

Sampling was with a two-stage cluster. In the first stage, two wards were randomly selected in each zone, and in the second stage, two villages were randomly selected from each of the six wards (n = 12). In each of the 12 villages, all women who had been pregnant during the previous two years were selected for interview using structured questionnaires. Women who were pregnant for the first time at the time of the data collection were excluded.

### Sample size

The crude birth rate in Tanzania is approximately 4% [2] which means 8% of the population is expected to have been pregnant or delivered in the past two years: this made 16,000 women eligible for the study. Assuming that 25% of the women were aware of obstetric danger signs, with a desired precision of 5% (95% confidence interval), a design effect of two and a non-participation rate of 10%, a total of 974 women were required for the study. The number of women selected for the study was 1 151 and of these, 33 (2.8%) were absent at the first and second visit and were regarded as non-respondents; thus, 1 118 women were interviewed.

### Data collection

The questionnaire, translated and back translated, Swahili to English to Swahili, to ensure relevance and accuracy. The questionnaire was then piloted in a similar district (Mkuranga) in the same region. The interviews were evaluated by the researchers and necessary changes made.

The questionnaire included socio-demographic characteristics including age, marital status, education level and occupation; pregnancy characteristics including number of deliveries, number of pregnancies and whether the women were pregnant or not at the time of the interview; experiences during their last pregnancy including whether they attended antenatal care, month of pregnancy booked for care, the number of visits made and if were informed of any risk or complication during antenatal care and danger signs of obstetric complications.

The antenatal cards used in their last pregnancy were available for 636 women and were reviewed for more information on the advice given to deliver in a hospital. Information on awareness of danger signs was collected by asking women if they knew any danger signs that may occur during pregnancy, delivery and after delivery separately in the same interview and those who knew danger signs were asked to mention them. Probing was used to elicit further responses.

The village leaders were informed of the research activities before data collection. House-to-house visits were made on the day of data collection. All women who had been pregnant in the past two years were identified and interviewed by pre-trained research assistants (nurse midwives).

Based on the recommendations of the national antenatal care guideline and the Safe Motherhood Initiative, a list of medically recognized life threatening obstetric signs were obtained from the women's responses. The list included vaginal bleeding during pregnancy and delivery, severe vaginal bleeding after delivery, anemia, swelling of lower limbs, fits of pregnancy, severe headache, high grade fever, child does not move, severe abdominal pain, awareness of fast heart beats, high blood pressure, prolonged labor, loss of consciousness and retained placenta.

### Data analysis

After data collection, responses for open-ended questions were reviewed, categorized, and coded for computerization. Data were entered with Epi Info and subsequently analyzed with SPSS. Awareness of danger signs of obstetric complication in this study was defined as the ability to mention at least one recognized danger sign during pregnancy, delivery or after delivery. Chi-square test was used to determine associations between categorical variables. The differences were deemed significant when p < 0.05. Bivariate logistic regression analysis was used to identify factors associated with awareness of obstetric danger signs. Variables significant in the bivariate analysis were then entered into a multivariate logistic regression analysis. The associations between awareness and each independent variable were estimated by odds ratio (OR) and 95% confidence interval (CI). A CI was considered statistically significant when the interval between the upper and lower values did not include one.

### Ethical approval

The Muhimbili University of Health and Allied Sciences (MUHAS) research and publication committee gave ethical clearance to conduct the study. Permission to conduct the study was obtained from Rufiji district and village authorities. The purpose of study, benefits, right to refuse participation, and liberty to refuse or leave the study at any time was explained to each participant before the interview. Verbal consent was regarded as sufficient to be included in the study. To ensure confidentiality, women's names were not written on the questionnaires.

## Results

### Socio-demographic and pregnancy characteristics

The total number of women interviewed was 1 118. Median age was 26 (Range: 15–45) and median parity was three (Range: 1–14). A majority of women were married/cohabiting (80%) and peasants (77%). Almost half (46%) of the women had completed primary education. One thousand one hundred (98%) of the women attended antenatal care at least once and the median number of visits was four (Range: 0–10).

### Awareness of danger signs

**Figure 1:**
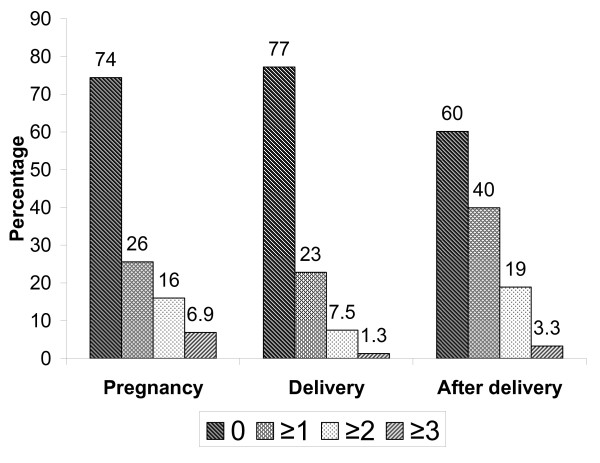
**Percent of women who knew 0, ≥ 1, ≥ 2, and ≥ 3 obstetric danger signs during pregnancy, delivery and after delivery**.

Five hundred and seventy one (51.1%) of the women knew at least one obstetric danger sign. The percentage of women who knew at least one danger sign related to pregnancy was 26%, in relation to delivery 23%, and to the period after delivery 40%. Few women knew three or more danger signs, especially for the delivery period (Figure 1).

One in four women recognized severe vaginal bleeding after delivery as a danger sign. Vaginal bleeding during pregnancy (9.6%) and delivery (13%) were mentioned as danger signs. Other danger signs known were anemia and seizures during pregnancy. Prolonged labor was known by only 1.5% of the women, while retained placenta was recognized by 8.0% (Table 1).

**Table 1:** Women's awareness of obstetric danger signs during pregnancy, during delivery and after delivery (N = 1118).

Obstetric danger sign	Awareness of					
	
	Pregnancy		Delivery		After delivery	
			
	n	%	n	%	n	%
Vaginal bleeding	107	9.6	149	13.3	259^a^	23.2
Lack of blood (Anemia)	97	8.7	28	2.5	83	7.4
Fits of pregnancy	78	7.0	103	9.2	116	10.4
Severe abdominal pain	60	5.4	19	1.7	52	4.7
High grade fever	56	5.0	7	0.6	32	2.9
High blood pressure	37	3.3	15	1.3	18	1.6
Severe headache	21	1.9	7	0.6	5	0.4
Loss of consciousness			8	0.7	25	2.7
Swelling of the lower limbs	77	6.9			21	1.9
Awareness of fast heart beat	18	1.6				
Child does not move	13	1.2				
Prolonged labor			17	1.5		
Retained placenta					89	8.0
Do not know any of the above	832	74.4	863	77.2	672	60.1

^a ^severe vaginal bleeding

### Factors associated with awareness of danger signs

There were no differences in the awareness of danger signs during pregnancy, during delivery or after delivery as related to age, educational level, number and place of deliveries, number of antenatal care visits and woman informed of a risk/complication during antenatal care (p < 0.05) (Table 2).

**Table 2:** Women's awareness of danger signs during pregnancy, during delivery and after delivery presented by socio-demographic, obstetric characteristics, experience in the last pregnancy, and antenatal care attendance (N = 1118).

	Awareness of danger signs during						
		
Socio-demographic and obstetric characteristic	Pregnancy		Delivery		After delivery		Total
				
	n	%	n	%	n	%	
*Age:*	p < 0.001		p < 0.001		p < 0.001		
-19	20	14.5	11	8.0	28	20.3	138
20–29	140	23.1	130	21.5	229	37.9	605
30–39	106	34.4	96	31.2	152	49.4	308
40-	20	29.9	18	26.9	37	55.2	67
*Marital status:*	p = 0.25		p = 0.85		p = 0.12		
Single	31	20.7	32	21.3	51	34.0	150
Married/Cohabiting	233	26.0	205	22.9	360	40.2	895
Divorced/Separated/Widowed	22	30.1	18	24.7	35	47.9	73
*Educational level:*	p < 0.001		p < 0.001		p < 0.001		
Not gone to school	78	17.5	68	15.2	150	33.6	446
Primary school incomplete	35	25.5	33	24.1	51	37.2	137
Primary school complete	158	30.7	145	28.2	231	44.9	515
Secondary school and above	15	75.0	9	45.0	14	70.0	20
*Occupation:*	p = 0.084		p = 0.44		p = 0.15		
Peasant	214	24.9	197	23.0	328	38.2	858
Housewife	19	19.4	17	17.3	41	41.8	98
Petty businesswomen	44	32.1	36	26.3	64	46.7	137
Other	9	36.0	5	20.0	13	52.0	25
*Number of deliveries*	p = 0.004		p = 0.002		p < 0.001		
1	43	19.3	32	14.3	62	27.8	223
2 – 4	134	24.5	132	24.1	208	38.0	548
5 -	109	31.4	91	26.2	176	50.7	347
*Place of delivery:*	p < 0.001		p < 0.001		p < 0.001		
Home/Roadside	74	19.6	57	15.1	125	33.1	378
Health institution	212	28.6	198	26.8	321	43.4	740
*Number of antenatal care visits*^*a*,*b*^:	p < 0.001		p < 0.001		p < 0.001		
-3	105	20.5	94	18.4	178	34.8	511
4-	171	29.9	150	26.3	254	44.5	571
*Month booked antenatal care*^*a*^:	p = 0.138		p = 0.007		p = 0.087		
-3	67	28.5	68	28.9	104	44.3	235
4-	214	24.7	182	21.0	338	39.1	865
*Informed of a risk/complication during antenatal care*^*a*^:	p < 0.001		p < 001		p = 0.003		
Yes	71	39.7	66	36.9	89	49.7	179
No	210	22.8	184	20.0	353	38.3	921
*Advised to deliver in hospital*^*c*^:	p = 0.15		p = 0.36		p = 0.20		
Yes	58	23.9	56	23.0	93	38.3	243
No	110	28.0	97	24.7	165	42.0	393

Note: P-value for χ^2 ^– test between those who did not know of any danger signs versus those who knew one or more danger sign.

^a ^18 women did not attend antenatal care

^b ^18 women did not know the number of antenatal care visits

^c ^482 women had no antenatal cards

The independent variables marital status, occupation, and advice to deliver in hospital were not associated with awareness of a danger sign during pregnancy, delivery and after delivery in the bivariate logistic regression analysis, and were thus not included in the multivariate logistic regression analysis. Having secondary education or higher increased the likelihood of awareness of obstetric danger signs six-fold (OR = 5.8; 95% CI: 1.8–19). Moreover, the likelihood of awareness of obstetric danger signs increased with age, number of deliveries, number of antenatal visits, when delivery was at a health institution, and when the mother had been informed of having a risk factor or complication during antenatal care. (Table 3)

**Table 3:** Bivariate and multivariate logistic regression analysis of the likelihood of knowing one or more danger sign during pregnancy, during delivery and after delivery.

Socio-demographic and obstetric characteristic	Aware of danger sign		Bivariate analysis		Multivariate analysis	
			
	Yes	No	OR	95% CI	OR	95% CI
*Age:*						
-19	41	97	1		1	
20–29	295	310	2.3	1.5–3.4	1.7	1.1–2.8
30–39	192	116	3.9	2.5–6.0	2.3	1.2–4.1
40-	43	24	4.2	2.3–7.9	2.3	1.2–5.2
*Marital status:*						
Single	68	82	1			
Married/Cohabiting	461	434	1.3	0.91–1.8		
Divorced/Separated/Widowed	42	31	1.6	0.93–2.9		
*Educational level:*						
Not gone to school	196	250	1		1	
Primary school incomplete	62	75	1.1	0.72–1.5	1.0	0.67–1.6
Primary school complete	297	218	1.7	1.3–2.2	1.7	1.3–2.3
Secondary school and above	16	4	5.1	1.7–15	5.8	1.8–19
*Occupation:*						
Peasant	430	428	1			
Housewife	47	51	0.9	0.60–1.4		
Petty businesswomen	80	57	1.3	0.67–2.8		
Other	14	11	1.4	0.97–2.0		
*Number of deliveries*						
1	84	139	1		1	
2 – 4	275	273	1.7	1.2–2.3	1.7	1.1–2.6
5 -	212	135	2.6	1.8–3.7	2.2	1.3–3.8
*Place of delivery:*						
Home/Roadside	168	210	1		1	
Health institution	403	337	1.5	1.2–1.9	1.4	1.1–1.9
*Number of antenatal care visits*^*a*,*b*^:						
-3	230	281	1		1	
4-	322	249	1.5	1.2–2.0	1.4	1.1–1.9
*Month booked antenatal care*^*a*^:						
-3	137	98	1		1	
4-	427	438	0.70	0.52–0.93	0.89	0.64–1.2
*Informed of a risk/complication during antenatal care*^*a*^:						
Yes	122	57	2.3	1.7–3.3	2.6	1.8–3.8
No	442	479	1		1	
*Advised to deliver in hospital*^*c*^:						
Yes	119	124	1			
No	207	186	1.2	0.84–1.6		

^a ^18 women did not attend antenatal care

^b ^18 women did not know the number of antenatal care visits

^c ^482 women had no antenatal cards

## Discussion

In this rural district in Tanzania, almost half of the women were not aware of any danger sign of obstetric complications which is the sign of low awareness. A higher level of education was the most important predictive factor for increased awareness of danger signs. Other factors associated with increased awareness included multiparity, age, more than four antenatal care visits, and woman informed of having a risk or complication during antenatal care visits.

Most women attended antenatal care where they should have been informed about danger signs. However, a limitation of this study is that women were not asked of the source of information of the danger signs. Therefore it is difficult to decide whether their knowledge come from the antenatal care, personal experiences or general awareness in the community. Moreover, despite choosing the women who had pregnancy in the last two years there could be still room for the recall bias of the experiences in their last pregnancy.

Women were more aware of danger signs occurring after delivery than during pregnancy and labor/delivery. Severe vaginal bleeding after delivery was the most recognized danger sign, and was mentioned twice as often as other signs, such as vaginal bleeding during pregnancy and delivery, anemia, and fits in pregnancy. Higher awareness of vaginal bleeding after delivery is also reported in a poor fishing community in Karachi, Pakistan [14]. The reason excessive vaginal bleeding after delivery is most commonly recognized as a danger sign may be that it is the most visible sign and the most common cause of maternal death immediately after delivery [5,15]. Furthermore, the mean interval from the onset of severe bleeding to death is two hours in contrast to an average of 12 hours for bleeding during pregnancy and delivery [15].

Few women were aware of prolonged labor as an obstetric danger sign despite its association with both maternal and fetal morbidity and mortality. In a study in The Gambia, involving urban and rural women attending antenatal care, prolonged labor was not recognized as a danger sign [16], a similar finding was reported from Malawi [17]. However, a study in Pakistan [14] reported that 23% of women are aware of this danger sign. The difference in knowledge observed is difficult to explain but it may be due to how interviews were conducted, whether prolonged labour is one among danger signs women are counseled during antenatal care or perception of prolonged labour in these culturally different areas.

Increased awareness among older and multiparous women may be related to their own experiences of pregnancy or events in the community. Women's own experience was an important source of information, as women who had experienced obstetric risk or complication in their last pregnancy were more aware of danger signs. This implied that young women in their first pregnancy may need more consideration when providing counseling and health education.

Women who had completed primary education had higher awareness of danger signs than women with incomplete or no formal education. Better education is associated with enlightenment and awareness of different health conditions although exposure to information is crucial [17]. Studies in Tanzania and elsewhere indicate that a higher level of education is associated with lower maternal mortality [4,18,19] whereas other studies have shown no association [20,21]. Despite these conflicting results, we still believe that women's education is important for understanding health messages and to be able to make decisions regarding their health and care. Introducing appropriate Safe Motherhood information in primary schools to girls before they become pregnant may further improve women understanding of health messages including awareness of danger signs of obstetric complication [22].

In this study, 98.4% of the women had attended antenatal care. Women who made four or more antenatal care visits were more aware of danger signs, independent of gestational age at booking. It is worth noting that women advised to deliver in hospital due to risk identified during antenatal care were not more aware of danger signs than those not advised. Provision of information aimed at increasing awareness of risk factors and danger signs in pregnancy is a challenge to antenatal programs and the difficulties involved should not be underestimated. A recent Cochrane [23] review failed to find high quality evidence for the benefit of antenatal education for child birth. Furthermore, a literature review of qualitative studies concluded that interaction between patient and nurse has a complex and multifaceted nature [24]. Studies from The Gambia, Nepal, Tanzania and Zimbabwe, report that less than three minutes are spent on individual counseling per consultation in antenatal clinics [16,25-27], whereas, simulation of FANC in Tanzania shows that the necessary time to provide appropriate information is 15 minutes [26]. It is recommended that the sociocultural aspects should be taken into account in modern concepts of information, education and communication (IEC) [28].

In addition to the antenatal and postnatal care counseling, other sources of information including community-based radio messages and educational sessions for women's groups, husbands, mothers in-law, and other family members, who play an important role in the decision making process, require strengthening [7,29]. Further studies on the quality of counseling on danger signs and utilization of health services, and appropriate training modalities for health workers are needed. Qualitative approaches, such as in-depth interviews, can be used to explore how women perceive the information given.

## Conclusion

In this study, although majority of the women attended antenatal care but generally had low awareness of danger signs of obstetric complications. Better awareness of danger signs was strongly associated with higher level of education of the woman.

We recommend the following in order to increase awareness of danger signs of obstetrical complications: To improve quality of counseling to women on health messages especially danger signs of obstetric complication, and involving husbands and other family members in antenatal and postnatal care; to use radio messages and educational sessions targeting the whole community and to intensify provision of formal education as emphasized in the second millennium development goal to enable women better understand information given.

## Competing interests

The authors declare that they have no competing interests.

## Authors' contributions

ABP participated in the design of the study, data collection, performed statistical analysis and drafted the first manuscript. DPU participated in design of the study and data collection. AC participated in design of the study and helped to draft the manuscript. GL participated in design of the study and reviewed the manuscript. LN participated in design of the study, helped to performed statistical analysis and interpretation. ED conceived of the study, participated in the design and helped to draft the manuscript. All authors read and approved the final manuscript.

## Pre-publication history

The pre-publication history for this paper can be accessed here:


